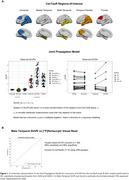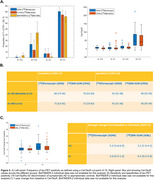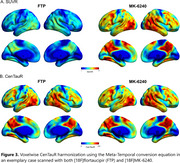# External Validation of Joint Propagation Model‐Based Tau PET CenTauR units

**DOI:** 10.1002/alz70862_109722

**Published:** 2025-12-23

**Authors:** Alexis Moscoso, Antoine Leuzy, Lars Lau Raket, Victor L. Villemagne, Gregory Klein, Matteo Tonietto, Emily Olafson, Suzanne L. Baker, Ziad S. Saad, Santiago Bullich, Brian J Lopresti, Sandra Sanabria, Olivia Lutz, Mercè Boada, Tobey J. Betthauser, Arnaud Charil, Emily C. Collins, Jessica Collins, Roger N Gunn, Makoto Higuchi, Eric D. Hostetler, R. Matthew Hutchison, Leonardo Iaccarino, Philip S. Insel, Michael C. Irizarry, Clifford R. Jack, William J. Jagust, Keith A. Johnson, Sterling C Johnson, Yashmin Karten, Marta Marquié, Sulantha Mathotaarachchi, Mark A. Mintun, Rik Ossenkoppele, Qi Huang, Xiaxie Mao, Johannes Gnörich, Ioannis Pappas, Ronald Petersen, Konstantinos Chiotis, Gil D. Rabinovici, Pedro Rosa‐Neto, Christopher G Schwarz, Ruben Smith, Andrew W. Stephens, Alex Whittington, Maria C. Carrillo, Michael Pontecorvo, Samantha Budd Haeberlein, Billy Dunn, Hartmuth C. Kolb, Diane Stephenson, Nadine Tatton, Matthias Brendel, Fang Xie, Christopher C. Rowe, Oskar Hansson, Vincent Dore

**Affiliations:** ^1^ 1Nuclear medicine department and Molecular Imaging Group, Instituto de Investigación Sanitaria de Santiago de Compostela, Santiago de Compostela, Galicia Spain; ^2^ Critical Path for Alzheimer’s Disease (CPAD) Consortium, Critical Path Institute, Tucson, AZ USA; ^3^ Critical Path Institute, Tucson, AZ USA; ^4^ Eli Lilly and Company, Indianapolis, IN USA; ^5^ The University of Pittsburgh, Pittsburgh, PA USA; ^6^ Roche Pharma Research and Early Development, FHoffmann‐La RocheLtd, Basel Switzerland; ^7^ Roche Pharma Research and Early Development, Neuroscience and Rare Diseases Biomarkers, F. Hoffmann‐La Roche Ltd., Basel Switzerland; ^8^ Genentech, Inc., San Francisco, CA USA; ^9^ Lawrence Berkeley National Laboratory, Berkeley, CA USA; ^10^ Johnson & Johnson Innovative Medicine, San Diego, CA USA; ^11^ Life Molecular Imaging GmbH, Berlin Germany; ^12^ University of Pittsburgh, Pittsburgh, PA USA; ^13^ Genentech, San Fransisco, CA USA; ^14^ University of Chicago, Chicago, IL USA; ^15^ Genentech, Inc, San Francisco, CA USA; ^16^ Ace Alzheimer Center Barcelona‐Universitat Internacional de Catalunya, Barcelona Spain; ^17^ University of Wisconsin‐Madison School of Medicine and Public Health, Madison, WI USA; ^18^ Eisai Inc., Nutley, NJ USA; ^19^ Biogen, Cambridge, MA USA; ^20^ Xing Imaging – A Mitro Company, London UK; ^21^ National Institutes for Quantum Science and Technology, Chiba Japan; ^22^ Merck Sharp & Dohme LLC, West Point, PA USA; ^23^ University of California, San Francisco, San Francisco, CA USA; ^24^ Eisai Inc., Woodcliff Lake, NJ USA; ^25^ Mayo Clinic, Rochester, MN USA; ^26^ Neuroscience Department, University of California, Berkeley, Berkeley, CA USA; ^27^ Center for Alzheimer’s Research and Treatment, Department of Neurology, Brigham and Women’s Hospital, Harvard Medical School, Boston, MA USA; ^28^ Department of Medicine, University of Wisconsin‐Madison School of Medicine and Public Health, Madison, WI USA; ^29^ Ace Alzheimer Center Barcelona, Barcelona Spain; ^30^ Enigma Biomedical Group, Knoxville, TN USA; ^31^ Amsterdam University Medical Center, Amsterdam Netherlands; ^32^ Clinical Memory Research Unit, Department of Clinical Sciences, Lund University, Lund Sweden; ^33^ Department of Nuclear Medicine & PET Center, Huashan Hospital, Fudan University, Shanghai, Shanghai China; ^34^ Department of Nuclear Medicine & PET Center, Huashan Hospital, University, Shanghai China; ^35^ LMU University Hospital, Munich Germany; ^36^ Laboratory of Neuro Imaging, Stevens Neuroimaging and Informatics Institute, Keck School of Medicine, University of Southern California, Los Angeles, CA USA; ^37^ Memory and Aging Center, Weill Institute for Neurosciences, University of California San Francisco, San Francisco, CA USA; ^38^ UCSF Alzheimer's Disease Research Center, San Francisco, CA USA; ^39^ McConnell Brain Imaging Centre, Montreal Neurological Institute, McGill University, Montreal, QC Canada; ^40^ Department of Radiology, Mayo Clinic, Rochester, MN USA; ^41^ Clinical Memory Research Unit, Lund University, Malmö, Skåne Sweden; ^42^ Invicro, London UK; ^43^ Alzheimer's Association, Chicago, IL USA; ^44^ Enigma Biomedical Group, Knovxille, TN USA; ^45^ Senior advisor to CPAD Consortium, Critical Path Institute, Tucson, AZ USA; ^46^ Department of Nuclear Medicine, University Hospital, LMU Munich, Munich, Bavaria Germany; ^47^ Huashan Hospital, Fudan University, Shanghai, Shanghai China; ^48^ The Australian Dementia Network (ADNeT), Melbourne, VIC Australia; ^49^ Clinical Memory Research Unit, Department of Clinical Sciences Malmö, Lund University, Lund Sweden; ^50^ The Australian e‐Health Research Centre, Commonwealth Scientific and Industrial Research Organisation, Brisbane, QLD Australia

## Abstract

**Background:**

The Joint Propagation Model (JPM)‐based CenTauR scale was recently introduced to harmonize tau‐PET quantification across different radiotracers. This study examined how CenTauR harmonization enhances the comparability of tau‐PET quantification across matched cohorts using different tracers. The evaluation focused on three key aspects: (1) comparing tau‐PET positivity rates as defined by CenTauR, (2) evaluating its diagnostic accuracy for symptomatic AD, and (3) analyzing longitudinal changes in tau‐PET rates over time.

**Method:**

The JPM, developed by the CPAD‐led Tau PET Harmonization Working Group (Leuzy et al., Alzheimers Dement. 2024; Figure 1A), models relationships between anchor point subjects and head‐to‐head tau‐PET SUVR data onto the CenTauR scale, providing conversion equations for multiple tracers. JPM equations were applied to [¹⁸F]flortaucipir SUVR (Meta‐temporal ROI) data from 561 cognitively impaired participants in the ADNI and OASIS‐3 studies. Using ROC analysis, we determined the CenTauR cut‐off for positivity (T+) that maximized the Youden index for discriminating between FDA‐approved positive/negative visual reads. Three separate cohorts, scanned using [¹⁸F]flortaucipir (ADNI), [¹⁸F]MK‐6240 (CPAS), and [¹⁸F]RO‐948 (BioFINDER‐2), were matched 1:1 by age, MMSE, amyloid‐β, and clinical diagnosis. CenTauR‐based metrics were compared across these cohorts.

**Result:**

A cut‐off of 18 CenTauRs was found to optimally classify [^18^F]flortaucipir PET visual reads (Figure 1B). The matching procedure identified 1089 participants (363 per cohort). The frequency of T+ using the 18 CenTauRs cut‐off for the Meta‐Temporal ROI was highly comparable across tracers across groups, except for the Aβ‐positive cognitively unimpaired group, where variability was more pronounced due to a smaller sample size (Figure 2A). Similarly, tau‐PET discriminative accuracy for symptomatic AD vs controls remained similar across [^18^F]flortaucipir and [^18^F]MK‐6240 (Figure 2B). In a separate sample of 212 matched participants from ADNI ([¹⁸F]flortaucipir) and AIBL ([¹⁸F]MK‐6240) with baseline and 1‐year follow‐up tau‐PET scans, 1‐year change in CenTauRs was comparable (Figure 2C). An exploratory voxelwise transformation, utilizing the Meta‐Temporal conversion equation in a representative case scanned with both [¹⁸F]flortaucipir and [¹⁸F]MK‐6240, demonstrates the potential for voxelwise CenTauR harmonization (Figure 3).

**Conclusion:**

These analyses suggest that CenTauR harmonization increases the comparability of tau‐PET data acquired with different tracers. Additional validation analyses with larger cohorts including other radiotracers ([¹⁸F]PI‐2620) are underway.